# Supplementary material for: From Loss of Control to Social Exclusion: ERP Effects of Preexposure to a Social Threat in the Cyberball Paradigm
Source: Brain Sci. 2022 Sep 10;12(9):1225. doi: 10.3390/brainsci12091225 (PMC9496925; doi:10.3390/brainsci12091225)
Supplement: Supplementary file 1 [file brainsci-12-01225-s001.zip › brainsci-1900137-supplementary.pdf]

Supplementary Materials

From Loss-of-Control to Social Exclusion: ERP Correlates of a Preexposure Effect in the Cyberball Paradigm

Xu Fang[1], Yu-Fang Yang[1], Rudolf Kerschreiter[2], Michael Niedeggen[1*]

1 Division of Experimental Psychology and Neuropsychology, Department of Education and Psychology, Freie Universität Berlin, Berlin, Germany, 2 Division of Social, Organizational, and Economic Psychology, Department of Education and Psychology, Freie Universität Berlin, Berlin, Germany
* Correspondence: michael.niedeggen@fu-berlin.de

The document contains the result of the analysis of additional dependent variables not mentioned in detail in the main manuscript.

S1: Additional Self Reports

S2: ERP effect of frontal electrode clusters

S3: Split-half analysis of the ERP data

S4: Alpha asymmetry

S1: Self-reports

Table S1. Additional self-reported data. Control: the control group; $EG1_{disc}$: the first experimental group (the preexposure pro-vided but discontinued); $EG2_{cont}$: the second experimental group (the preexposure pro-vided and continued). *M*: mean; *SD*: standard deviation; *CI*: 95% confidence interval.

	**Control** (n = 23)		**EG1$_{disc}$** (n = 23)		**EG2$_{cont}$** (n = 24)	
	M (SD)	*CI*	*M (SD)*	*CI*	*M (SD)*	*CI*
Estimated intervention in block1 (%)	8.43 (10.88)	[3.73, 13,14]	23.96 (18.83)	[15.81, 32.10]	18.71 (13.86)	[12.86, 24.56]
Estimated intervention in block2 (%)	22.35 (26.12)	[11.05, 33.64]	19.70 (22.08)	[10.15, 29.25]	33.13 (21.86)	[23.89, 42.36]
Personal power	-.61 (.83)	[-.97, -.25]	.28 (1.10)	[-.19, .76]	-.56 (.91)	[-.95, -.18]

Results of statistical analysis:

Estimated frequency of intervention

The main effect of 'group' in block 1: F (2, 67) = 6.487, p = .003, η_p^2 = .162

Post-hoc analysis: $EG1_{disc}$ vs Control: F (1, 44) = 11.717, p = .001, η_p^2 = .210;

$EG2_{cont}$ vs Control: F (1, 45) = 7.945, p = .007, η_p^2 = .150;

$EG1_{disc}$ vs $EG2_{cont}$ F (1, 45) = 1.191, p = .281, η_p^2 = .026

Conclusion: Manipulation of preexposure was effective in the experimental groups

Rating on the scale 'Personal Power'

The main effect of 'group': F (2, 67) = 6.447, p = .003, η_p^2 = .161

Post-hoc analysis: $EG1_{disc}$ vs Control: F (1, 44) = 9.712, p = .003, η_p^2 = .181;

$EG2_{cont}$ vs Control: F (1, 45) = .033, p = .857, η_p^2 = .001;

$EG1_{disc}$ vs $EG2_{cont}$: F (1, 45) = 8.283, p = .006, η_p^2 = .155

Conclusion: The rating of personal power is increased by the offset of interventions

S2: ERP results

Frontal P3 amplitude

	**Control** (n = 23)		**EG1**$_{disc}$ (n = 23)		**EG2**$_{cont}$ (n = 24)	
	M (SD)	*CI*	*M (SD)*	*CI*	*M (SD)*	*CI*
P3 amplitude (300–400ms) in block1 (μV)	2.97 (2.45)	[2.00, 3.93]	2.65 (1.96)	[1.69, 3.62]	1.01 (2.49)	[.07, 1.96]
P3 amplitude (300–400ms) in block2 (μV)	4.57 (2,46)	[3.71, 5.43]	4.68 (1.87)	[3.82, 5.55]	1.77 (1.82)	[.93, 2.62]
P3 amplitude (400–500ms) in block1 (μV)	.49 (1.95)	[-.48, 1.45]	1.63 (2.30)	[.66, 2.60]	-.49 (2.66)	[-1.44, -.46]
P3 amplitude (400–500ms) in block2 (μV)	2.19 (2.11)	[1.28, 3.10]	3.13 (2.05)	[2.22, 4.04]	-.27 (2.39)	[-1.62, .63]

Table S2. The frontal P3 amplitude was separated for group assignment (preexposure: control, EG1$_{disc}$**, and EG2**$_{cont}$**).** Control: the control group; EG1$_{disc}$: the first experimental group (the preexposure pro-vided but discontinued); EG2$_{cont}$: the second experimental group (the preexposure pro-vided and continued). *M*: mean; *SD*: standard deviation; *CI*: 95% confidence interval.

Analysis of effects in the early time range (300 – 400ms)

Effect of 'block': F (1, 67) = 22.334, p < .001, η_p^2 = .250

Interaction 'block' x 'group': F (2, 67) = 1.466, p = .238, η_p^2 = .042

Conclusion: The frontal P3 is enhanced by the transition of social exclusion, but the effect is not modulated by group assignment.

Analysis of the effects in the late time range (400 – 500ms)

Effect of 'block': F (1, 67) = 13.239, p = .001, η_p^2 = .165

Interaction 'block' x 'group': F (2, 67) = 2.206, p = .118, η_p^2 = .062.

Conclusion: The frontal P3 is enhanced by the transition of social exclusion, but the effect is not modulated by group assignment.

S3: Split-half analysis: adaptation effects within an experimental block

Table S3. The parietal P3 amplitude was split into two halves for each block in three groups (preexposure: control, $EG1_{disc}$, and $EG2_{cont}$). Control: the control group; $EG1_{disc}$: the first experimental group (the preexposure pro-vided but discontinued); $EG2_{cont}$: the second experimental group (the preexposure pro-vided and continued). *M*: mean; *SD*: standard deviation; *CI*: 95% confidence interval.

	**Control** (*n* = 23)		**$EG1_{disc}$** (*n* = 23)		**$EG2_{cont}$** (*n* = 24)	
	M (SD)	*CI*	*M (SD)*	*CI*	*M (SD)*	*CI*
Half 1 (300-400ms) in block1 (μV)	5.71 (.64)	[4.45, 6.98]	4.90 (.64)	[3.64,6.17]	5.65 (.62)	[4.41, 6.89]
Half 2 (300-400ms) in block1 (μV)	5.02 (.55)	[3.91, 6.12]	4.94 (.55)	[3.83, 6.04]	5.52 (.54)	[4.43, 6.60]
Half 1 (400-500ms) in block1 (μV)	4.42 (.67)	[3.10, 5.74]	3.37 (.66)	[2.05, 4.69]	3.80 (.65)	[2.51, 5.09]
Half 2 (400-500ms) in block1 (μV)	3.57 (.54)	[2.48, 4.66]	3.85 (.54)	[2.77, 4.94]	4.00 (.53)	[2.93, 5.05]
Half 1 (300-400ms) in block2 (μV)	7.68 (.68)	[6.32, 9.04]	6.16 (.68)	[4.80, 7.53]	5.48 (.67)	[4.14, 6.81]
Half 2 (300-400ms) in block2 (μV)	6.57 (.68)	[5.20, 7.93]	5.71 (.68)	[4.35, 7.08]	5.36 (.67)	[4.03, 6.70]
Half 1 (400-500ms) in block2 (μV)	6.32 (.66)	[5.01, 7.64]	5.02 (.66)	[3.71, 6.33]	3.69 (.64)	[2.41, 4.98]
Half 2 (400-500ms) in block2 (μV)	6.37 (.69)	[5.00, 7.74]	4.55 (.69)	[3.18, 5.92]	4.03 (.67)	[2.69, 5.38]

Analysis of effects in the early time range (300 – 400ms) in block 1

Effect of 'segment': F (1, 67) = .423, p = .518, η_p^2 = .006

Effect of 'group': F (2, 67) = .506, p = .605, η_p^2 = .015

Interaction 'block' x 'group': F (2, 67) = .291, p = .748, η_p^2 = .009

Conclusion: There is no significant change (decrease) in parietal P3 amplitudes (300 – 400ms) in block 1 in this Cyberball.

Analysis of the effects in the late time range (400 – 500ms) in block 1

Effect of 'segment': F (1, 67) = .022, p = .884, η_p^2 = .000

Effect of 'group': F (2, 67) = .159, p = .853, η_p^2 = .005

Interaction 'block' x 'group': F (2, 67) = 1.070, p = .349, η_p^2 = .031

Conclusion: There is no significant decrease in parietal P3 amplitudes (400 – 500ms) in block 1 in this Cyberball.

Analysis of effects in the early time range (300 – 400ms) in block 2

Effect of 'segment': F (1, 67) = 2.050, p = .157, η_p^2 = .030

Effect of 'group': F (2, 67) = 2.203, p = .118, η_p^2 = .062

Interaction 'block' x 'group': F (2, 67) = .568, p = .569, η_p^2 = .017

Conclusion: There is also no significant decrease in parietal P3 amplitudes (300 – 400ms) in block 2 in this Cyberball.

Analysis of the effects in the late time range (400 – 500ms) in block 2

Effect of 'segment': F (1, 67) = .010, p = .922, η_p^2 = .000

Effect of 'group': F (2, 67) = 4.225, p = .019, η_p^2 = .112

Interaction 'block' x 'group': F (2, 67) = .607, p = .548, η_p^2 = .018

Conclusion: There is also no significant decrease in parietal P3 amplitudes (400 – 500ms) in block 2 in this Cyberball.

S4: Alpha Asymmetry

Analysis of frontal alpha asymmetry

The processing steps of alpha asymmetry analysis are as follows: firstly, data were offline-filtered (0.3 to 30 Hz, 24 dB/Oct), segmented based on manual settings for the whole block1 and block2, then segmented again according to division in equal-sized segments (epoch length: 1000 ms; overlap 500ms), and then baseline-corrected (0 to 1000 ms). If ocular artifacts (EOG > 50 μV, F3 < 25 μV or F4 < 25 μV) occurred in the individual segment, they were automatically excluded from the analysis. Next, alpha power from frontal electrodes (F3, F4) was extracted by fast Fourier transform using a Hamming window with the alpha band (8 – 13 Hz) separately for the first 100 s and the last 100 s in block 2. Finally, asymmetry scores were obtained by subtracting the natural log of alpha power of the left frontal site (F3) from the corresponding value on the right electrode (F4).

Table S4. The alpha band (8 – 13 Hz) was separately for the first 100 s and the last 100 s in block 2 in three groups (preexposure: control, $EG1_{disc}$, and $EG2_{cont}$). Control: the control group; $EG1_{disc}$: the first experimental group (the preexposure pro-vided but discontinued); $EG2_{cont}$: the second experimental group (the preexposure pro-vided and continued). *M*: mean; *SD*: standard deviation; *CI*: 95% confidence interval.

	**Control** (*n* = 23)		**$EG1_{disc}$** (*n* = 23)		**$EG2_{cont}$** (*n* = 24)	
	M (SD)	*CI*	*M (SD)*	*CI*	*M (SD)*	*CI*
First 100 s in block2 (μV)	.11 (.15)	[-.05, .07]	.03 (.13)	[-.03, .09]	.04 (.13)	[.-.02, .09]
Last 100 s in block2 (μV)	.006 (.15)	[-.05, .06]	.03 (.11)	[-.03, .08]	.03 (.12)	[-.03, .08]

Results of analysis:

Frontal cortical activity in block 2

3 x 2 ANOVA with the between-factor 'preexposure' (Control vs $EG1_{disc}$ vs $EG2_{cont}$) and the within-factor 'segment' (first 100 s vs last 100 s).

Main effect 'group': F (2, 67) = .217, p = .805, η_p^2 = .006

Main effect 'segment': F (1, 67) = 1.214, p = .274, η_p^2 = .018

Interaction: F (2, 67) = .051, p = .95, η_p^2 = .002

Conclusion: The different preexposure conditions did not affect the alpha asymmetry

Table S5. The German questionnaire was used in this study [5, 6].

Code:

Im Folgenden finden Sie eine Reihe von Aussagen, mit denen man **Gefühle** beschreiben kann. Bitte geben Sie für jede Aussage an, ob diese Gefühle **im ersten oder im zweiten Block stärker ausgeprägt** waren.

Sie haben folgende Skala zur Verfügung:							
In **Block 1** (Spiel auf der **Wiese**)<br>sehr viel stärker +++<br>viel stärker ++<br>etwas stärker +<br>als in **Block 2** / =<br>in beiden Blöcken gleich / In **Block 2** (Spiel auf dem **Strand**)<br>+ etwas stärker<br>++ viel stärker<br>+++ sehr viel stärker<br>als in **Block 1**	**Stärker in Block 1 (Wiese)** > Block 2			=	**Stärker in Block 2 (Strand)** > Block 1		
1. Ich fühlte mich „unverbunden“ mit den anderen.	+++	++	+	0	+	++	+++
2. Ich fühlte mich zurückgewiesen.	+++	++	+	0	+	++	+++
3. Ich fühlte mich wie ein Außenseiter.	+++	++	+	0	+	++	+++
4. Ich fühlte mich mächtig.	+++	++	+	0	+	++	+++
5. Ich hatte Kontrolle über den Ablauf der Interaktion.	+++	++	+	0	+	++	+++
6. Ich fühlte mich überlegen.	+++	++	+	0	+	++	+++
7. Ich fühlte mich unabhängig.	+++	++	+	0	+	++	+++
8. Ich fühlte mich frei von anderen.	+++	++	+	0	+	++	+++
9. Ich fühlte mich gut.	+++	++	+	0	+	++	+++
10. Ich fühlte mich schlecht.	+++	++	+	0	+	++	+++
11. Ich fühlte mich freundlich.	+++	++	+	0	+	++	+++
12. Ich fühlte mich unfreundlich.	+++	++	+	0	+	++	+++
13. Ich fühlte mich ärgerlich.	+++	++	+	0	+	++	+++
14. Ich fühlte mich traurig.	+++	++	+	0	+	++	+++
15. Ich fühlte mich angespannt.	+++	++	+	0	+	++	+++
16. Ich fühlte mich entspannt.	+++	++	+	0	+	++	+++
17. Wenn das Spiel fair verläuft, also wenn der Ball gleich häufig zu jeder Person geworfen wird, bekommt (bei drei Spielern insgesamt) jeder Spieler 33% der Würfe. Wie viel Prozent der Würfe haben Sie bekommen?	…in Block1? __%				…in Block2? ___%		
18. Wie Sie wissen, gab es im Spiel einen „Supervisor“, der in manchen Spielen entscheiden konnte, wo ihr Ballwurf landet. Wie viel Prozent Ihrer Ballwürfe wurden kontrolliert?	…in Block1? __%				…in Block2? __%		

Table S6. The English translation of the German questionnaire above.

Code:

<table>
<tr><td colspan="8">In the following, you find a series of statements related to your feelings during the ball-throwing game. Please provide a rating of whether the feeling was more expressed in the first or in the second block of the game.</td></tr>
<tr><td>Here is the scale you can use to rate the expression of the feeling:

In Block 1 (Game on a Meadow): Much stronger +++; stronger ++; slightly stronger +; as compared to Block 2

=: Comparable in both blocks

In Block 2 (Game at the beach): + Much stronger; ++ stronger; +++ slightly stronger; As compared to Block 1</td><td colspan="3">Stronger in Block 1 (Meadow)
>
Block 2</td><td>=</td><td colspan="3">Stronger in Block 2 (Beach)
>
Block 1</td></tr>
<tr><td>1. I felt disconnected.</td><td>+++</td><td>++</td><td>+</td><td>0</td><td>+</td><td>++</td><td>+++</td></tr>
<tr><td>2. I felt rejected.</td><td>+++</td><td>++</td><td>+</td><td>0</td><td>+</td><td>++</td><td>+++</td></tr>
<tr><td>3. I felt like an outsider.</td><td>+++</td><td>++</td><td>+</td><td>0</td><td>+</td><td>++</td><td>+++</td></tr>
<tr><td>4. I felt powerful.</td><td>+++</td><td>++</td><td>+</td><td>0</td><td>+</td><td>++</td><td>+++</td></tr>
<tr><td>5. I felt I had control over the course of the game.</td><td>+++</td><td>++</td><td>+</td><td>0</td><td>+</td><td>++</td><td>+++</td></tr>
<tr><td>6. I felt I had the ability to significantly alter events.</td><td>+++</td><td>++</td><td>+</td><td>0</td><td>+</td><td>++</td><td>+++</td></tr>
<tr><td>7. I felt independent.</td><td>+++</td><td>++</td><td>+</td><td>0</td><td>+</td><td>++</td><td>+++</td></tr>
<tr><td>8. I felt free from other people.</td><td>+++</td><td>++</td><td>+</td><td>0</td><td>+</td><td>++</td><td>+++</td></tr>
<tr><td>9. I felt good.</td><td>+++</td><td>++</td><td>+</td><td>0</td><td>+</td><td>++</td><td>+++</td></tr>
<tr><td>10. I felt bad.</td><td>+++</td><td>++</td><td>+</td><td>0</td><td>+</td><td>++</td><td>+++</td></tr>
<tr><td>11. I felt pleasant.</td><td>+++</td><td>++</td><td>+</td><td>0</td><td>+</td><td>++</td><td>+++</td></tr>
<tr><td>12. I felt unpleasant.</td><td>+++</td><td>++</td><td>+</td><td>0</td><td>+</td><td>++</td><td>+++</td></tr>
<tr><td>13. I felt annoyed.</td><td>+++</td><td>++</td><td>+</td><td>0</td><td>+</td><td>++</td><td>+++</td></tr>
<tr><td>14. I felt sad.</td><td>+++</td><td>++</td><td>+</td><td>0</td><td>+</td><td>++</td><td>+++</td></tr>
<tr><td>15. I felt tense.</td><td>+++</td><td>++</td><td>+</td><td>0</td><td>+</td><td>++</td><td>+++</td></tr>
<tr><td>16. I felt relaxed.</td><td>+++</td><td>++</td><td>+</td><td>0</td><td>+</td><td>++</td><td>+++</td></tr>
<tr><td>17. In case of a fair game, you will get the ball as frequently as your co-players. In the case of three players, this would be 33% of the ball throws. How frequently did you get the ball? Please estimate the frequency (in percentage).</td><td colspan="4">…in Block1? __%</td><td colspan="3">…in Block2? ___%</td></tr>
<tr><td>18. You probably remember the ‘supervisor’ who might get active in some games, and who decided on the recipient of a ball throw. How often did the supervisor control your ball throws? Please estimate the frequency (in percentage).</td><td colspan="4">…in Block1? __%</td><td colspan="3">…in Block2? __%</td></tr>
</table>

References

1. Kawamoto, T.; Nittono, H.; Ura, M. Cognitive, affective, and motivational changes during ostracism: an ERP, EMG, and EEG study using a computerized cyberball task. *Neurosci. J.* **2013**, 2013, https://doi.org/10.1155/2013/304674
2. Themanson, J. R.; Khatcherian, S. M.; Ball, A. B.; Rosen, P. J. An event-related examination of neural activity during social interactions. *Soc. Cogn. Affect. Neurosci.* **2013**, 8, 727-733, https://doi.org/10.1093/scan/nss058
3. Schuck, K.; Niedeggen, M.; Kerschreiter, R. Violated expectations in the cyberball paradigm: Testing the expectancy account of social participation with ERP. *Front. Psychol.* **2018**, 1762, https://doi.org/10.3389/fpsyg.2018.01762
4. Harmon-Jones, E.; Gable, P. A. On the role of asymmetric frontal cortical activity in approach and withdrawal motivation: An updated review of the evidence. *Psychophysiology* **2018**, 55, e12879, https://doi.org/10.1111/psyp.12879
5. Jamieson, J. P.; Harkins, S. G.; Williams, K. D. Need threat can motivate performance after ostracism. *Pers. Soc. Psychol. Bull.* **2010**, 36, 690-702, https://doi.org/10.1177/0146167209358882
6. Gerber, J.; Chang, S.-H.; Reimel, H. Construct validity of Williams' ostracism needs threat scale. *Pers. Individ. Dif.* **2017**, 115, 50-53, https://doi.org/10.1016/j.paid.2016.07.008
